# (2*E*)-1-(2-Bromo­phen­yl)-3-(4-chloro­phen­yl)prop-2-en-1-one

**DOI:** 10.1107/S1600536810021562

**Published:** 2010-06-16

**Authors:** Jerry P. Jasinski, Ray J. Butcher, K. Veena, B. Narayana, H. S. Yathirajan

**Affiliations:** a Department of Chemistry, Keene State College, 229 Main Street, Keene, NH 03435-2001, USA; b Department of Chemistry, Howard University, 525 College Street NW, Washington, DC 20059, USA; c Department of Studies in Chemistry, Mangalore University, Mangalagangotri 574 199, India; d Department of Studies in Chemistry, University of Mysore, Manasagangotri, Mysore 570 006, India

## Abstract

In the title compound, C_15_H_10_BrClO, the dihedral angle between the mean planes of the benzene rings in the *ortho*-bromo- and *para*-chloro-substituted rings is 70.5 (6)°. The dihedral angles between the mean plane of the prop-2-en-1-one group and the mean planes of the benzene rings in the 4-chloro­phenyl and 2-bromo­phenyl rings are 14.9 (3) and 63.3 (8)°, respectively. In the crystal, inversion dimers linked by pairs of weak C—H⋯O interactions are observed as well as aromatic π–π stacking inter­actions.

## Related literature

For the radical quenching properties of the phenol groups present in many chalcones, see: Dhar (1981 ▶). For the anti­cancer activity of chalcones, see: Dimmock *et al.* (1999 ▶) and for their anti­malarial activity, see: Troeberg *et al.* (2000 ▶). For their non-linear optical properties, see: Sarojini *et al.* (2006 ▶). For related structures, see: Fun *et al.* (2008 ▶); Li *et al.* (2009 ▶); Ng *et al.* (2006 ▶); Teh *et al.* (2007 ▶); Yang *et al.* (2006 ▶), Jasinski *et al.* (2009 ▶, 2010 ▶). For bond-length data, see: Allen *et al.* (1987 ▶).
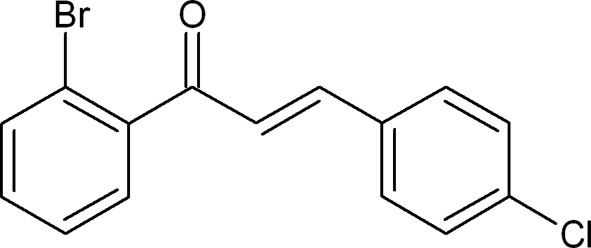


## Experimental

### 

#### Crystal data

C_15_H_10_BrClO
*M* *_r_* = 321.59
Monoclinic, 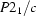

*a* = 5.7317 (6) Å
*b* = 9.3920 (7) Å
*c* = 23.6517 (18) Å
β = 91.231 (8)°
*V* = 1272.9 (2) Å^3^
*Z* = 4
Cu *K*α radiation
μ = 6.19 mm^−1^
*T* = 110 K
0.84 × 0.49 × 0.13 mm

#### Data collection

Oxford Diffraction Xcalibur Ruby Gemini R diffractometer
Absorption correction: multi-scan (*CrysAlis RED*; Oxford Diffraction, 2007 ▶) *T*_min_ = 0.039, *T*_max_ = 0.512
4362 measured reflections
2466 independent reflections
2275 reflections with *I* > 2σ(*I*)
*R*_int_ = 0.036

#### Refinement

*R*[*F* ^2^ > 2σ(*F* ^2^)] = 0.044
*wR*(*F* ^2^) = 0.126
*S* = 1.05
2466 reflections
163 parameters
H-atom parameters constrained
Δρ_max_ = 0.80 e Å^−3^
Δρ_min_ = −1.07 e Å^−3^

### 

Data collection: *CrysAlis PRO* (Oxford Diffraction, 2007 ▶); cell refinement: *CrysAlis PRO*; data reduction: *CrysAlis PRO*; program(s) used to solve structure: *SHELXS97* (Sheldrick, 2008 ▶); program(s) used to refine structure: *SHELXL97* (Sheldrick, 2008 ▶); molecular graphics: *SHELXTL* (Sheldrick, 2008 ▶); software used to prepare material for publication: *SHELXTL*.

## Supplementary Material

Crystal structure: contains datablocks global, I. DOI: 10.1107/S1600536810021562/zl2282sup1.cif

Structure factors: contains datablocks I. DOI: 10.1107/S1600536810021562/zl2282Isup2.hkl

Additional supplementary materials:  crystallographic information; 3D view; checkCIF report

## Figures and Tables

**Table 1:** Hydrogen-bond geometry (Å, °)

*D*—H⋯*A*	*D*—H	H⋯*A*	*D*⋯*A*	*D*—H⋯*A*
C14—H14*A*⋯O^i^	0.95	2.44	3.319 (4)	154

Symmetry code: (i) 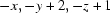.

**Fig. 1.**
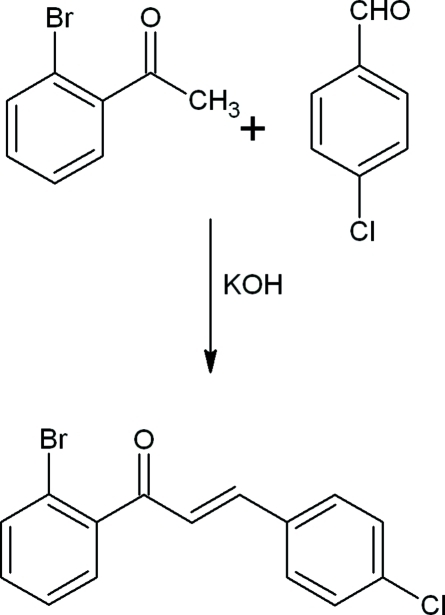
Reaction Scheme for the title compound.

**Fig. 2.**
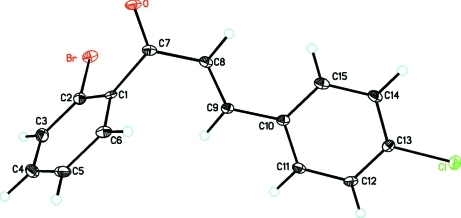
Molecular structure of the title compound, C15H10BrClO, showing the atom labeling scheme and 50% probability displacement ellipsoids.

**Fig. 3.**
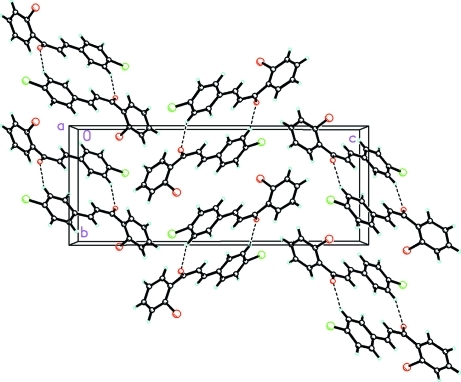
Packing diagram of the title compound, C15H10BrClO viewed down the a axis. Dashed lines indicate a weak C—H···O intermolecular hydrogen bond interaction which links the molecules into chains along the (011) direction.

## supplementary crystallographic information

### Comment

Chalcones, or 1,3-diaryl-2-propen-1-ones, belong to the flavonoid family. Chemically they consist of open-chain flavonoids in which the two aromatic rings are joined by a three-carbon α,β-unsaturated carbonyl system. A vast number of naturally occurring chalcones are polyhydroxylated in the aryl rings. The radical quenching properties of the phenol groups present in many chalcones have raised interest in using the compounds or chalcone rich plant extracts as drugs or food preservatives (Dhar, 1981). Chalcones have been reported to possess many useful properties, including anti-inflammatory, antimicrobial, antifungal, antioxidant, cytotoxic, anticancer activities (Dimmock *et al.*, 1999). Many chalcones have been described for their high antimalarial activity, probably as a result of Michael addition of nucleophilic species to the double bond of the enone (Troeberg *et al.*, 2000). Chalcones are finding applications as organic non-linear optical materials (NLO) due to their good SHG conversion efficiencies (Sarojini *et al.*, 2006). Hence, in continuation with our synthesis and crystal structure determinations of similar compounds (Jasinski *et al.*, 2009; Jasinski *et al.*, 2010)  and also owing to the importance of these flavanoid analogs, this new bromo-chloro substituted chalcone, C_15_H_10_BrClO, is synthesized and its crystal structure is reported.

The title compound, C_15_H_10_BrClO, is a chalcone with 4-chlorophenyl and 2-bromophenyl rings bonded to opposite sides of a propenone group (Fig. 2). The dihedral angle between mean planes of the benzene rings in the *ortho*-bromo and *para*-chloro substituted rings is 70.5 (6)°. The angle between the mean plane of the prop-2-ene-1-one group (C1/C7/O/C8) and the mean planes of the benzene rings in the 4-chlorophenyl (C10–CC15) and 2-bromophenyl rings (C1–C6) are 14.9 (3)° and 63.3 (8)°, respectively. Bond distances and angles are in normal ranges (Allen *et al.*, 1987). While no classical hydrogen bonds are present, a weak intermolecular C14—H14A···O interaction (Table 1) and weak π-π stacking interactions [Cg2_perp···Cg2_perp = 3.3466 (14) Å; slippage = 2.931 Å; 1-x, 2-y, 1-z] are observed which contribute to the stability of crystal packing (Fig. 3).

### Experimental

A 50% KOH solution was added to a mixture of 2-bromo acetophenone (0.01 mol, 1.99 g) and 4-chloro benzaldehyde (0.01 mol, 1.40 g) in 25 ml of ethanol (Fig. 1). The mixture was stirred for an hour at room temperature and the precipitate was collected by filtration and purified by recrystallization from ethanol. The single-crystal was grown from ethyl acetate by slow evaporation and the yield of the compound was 58% (m.p.368–370 K). Analytical data: Composition (%) found (Calculated): C: 55.97 (56.02); H: 3.09(3.13).

### Refinement

The H atoms were placed in geometrically idealized positions and constrained to ride on their parent atoms with C–H distances = 0.95Å and with *U*_iso_(H) = 1.18–1.22 *U*_eq_(C).

### Crystal data

**Table:**

C_15_H_10_BrClO	*F*(000) = 640
*M**_r_* = 321.59	*D*_x_ = 1.678 Mg m^−^^3^
Monoclinic, *P*2_1_/*c*	Cu *K*α radiation, λ = 1.54184 Å
Hall symbol: -P 2ybc	Cell parameters from 2738 reflections
*a* = 5.7317 (6) Å	θ = 4.7–74.2°
*b* = 9.3920 (7) Å	µ = 6.19 mm^−^^1^
*c* = 23.6517 (18) Å	*T* = 110 K
β = 91.231 (8)°	Plate, yellow
*V* = 1272.9 (2) Å^3^	0.84 × 0.49 × 0.13 mm
*Z* = 4	

### Data collection

**Table:**

Oxford Diffraction Xcalibur Ruby Gemini R diffractometer	2466 independent reflections
Radiation source: Enhance (Cu) X-ray Source	2275 reflections with *I* > 2σ(*I*)
graphite	*R*_int_ = 0.036
Detector resolution: 10.5081 pixels mm^-1^	θ_max_ = 74.2°, θ_min_ = 5.1°
ω scans	*h* = −6→6
Absorption correction: multi-scan (*CrysAlis RED*; Oxford Diffraction, 2007)	*k* = −11→10
*T*_min_ = 0.039, *T*_max_ = 0.512	*l* = −25→29
4362 measured reflections	

### Refinement

**Table:**

Refinement on *F*^2^	Primary atom site location: structure-invariant direct methods
Least-squares matrix: full	Secondary atom site location: difference Fourier map
*R*[*F*^2^ > 2σ(*F*^2^)] = 0.044	Hydrogen site location: inferred from neighbouring sites
*wR*(*F*^2^) = 0.126	H-atom parameters constrained
*S* = 1.05	*w* = 1/[σ^2^(*F*_o_^2^) + (0.0875*P*)^2^ + 2.2371*P*] where *P* = (*F*_o_^2^ + 2*F*_c_^2^)/3
2466 reflections	(Δ/σ)_max_ = 0.001
163 parameters	Δρ_max_ = 0.80 e Å^−^^3^
0 restraints	Δρ_min_ = −1.07 e Å^−^^3^

### Special details

**Table:**

Experimental. IR data (KBr) ν cm^-1^: 2837 cm^-1^, 2966 cm^-1^, (C—H al. str) 3061 cm^-1^, (C—H ar. str), 1655 cm^-1^ (C=O), 1584 cm^-1^ (C=C); 1254 cm^-1^ (C—O—C).
Geometry. All e.s.d.'s (except the e.s.d. in the dihedral angle between two l.s. planes) are estimated using the full covariance matrix. The cell e.s.d.'s are taken into account individually in the estimation of e.s.d.'s in distances, angles and torsion angles; correlations between e.s.d.'s in cell parameters are only used when they are defined by crystal symmetry. An approximate (isotropic) treatment of cell e.s.d.'s is used for estimating e.s.d.'s involving l.s. planes.
Refinement. Refinement of *F*^2^ against ALL reflections. The weighted *R*-factor *wR* and goodness of fit *S* are based on *F*^2^, conventional *R*-factors *R* are based on *F*, with *F* set to zero for negative *F*^2^. The threshold expression of *F*^2^ > σ(*F*^2^) is used only for calculating *R*-factors(gt) *etc*. and is not relevant to the choice of reflections for refinement. *R*-factors based on *F*^2^ are statistically about twice as large as those based on *F*, and *R*- factors based on ALL data will be even larger.

### Fractional atomic coordinates and isotropic or equivalent isotropic displacement parameters (Å^2^)

**Table:**

	*x*	*y*	*z*	*U*_iso_*/*U*_eq_	
Br	−0.27391 (6)	0.44265 (4)	0.639317 (15)	0.02097 (17)	
Cl	0.85133 (14)	0.88426 (9)	0.35152 (3)	0.0207 (2)	
O	−0.2348 (4)	0.7916 (3)	0.62618 (11)	0.0207 (5)	
C1	0.0767 (5)	0.6542 (3)	0.66371 (13)	0.0132 (6)	
C2	−0.0160 (6)	0.5245 (4)	0.68014 (13)	0.0168 (7)	
C3	0.0796 (7)	0.4490 (4)	0.72552 (15)	0.0228 (8)	
H3A	0.0157	0.3598	0.7360	0.027*	
C4	0.2681 (7)	0.5048 (4)	0.75513 (15)	0.0257 (8)	
H4A	0.3305	0.4549	0.7869	0.031*	
C5	0.3679 (6)	0.6328 (4)	0.73916 (14)	0.0230 (8)	
H5A	0.4999	0.6697	0.7593	0.028*	
C6	0.2715 (6)	0.7065 (4)	0.69309 (14)	0.0192 (7)	
H6A	0.3399	0.7937	0.6816	0.023*	
C7	−0.0380 (6)	0.7467 (3)	0.61912 (14)	0.0150 (6)	
C8	0.0907 (6)	0.7835 (4)	0.56835 (13)	0.0163 (6)	
H8A	0.0264	0.8560	0.5447	0.020*	
C9	0.2903 (5)	0.7243 (3)	0.55238 (13)	0.0139 (6)	
H9A	0.3548	0.6525	0.5763	0.017*	
C10	0.4194 (5)	0.7595 (3)	0.50142 (13)	0.0143 (6)	
C11	0.6205 (6)	0.6846 (4)	0.48865 (13)	0.0167 (7)	
H11A	0.6685	0.6077	0.5122	0.020*	
C12	0.7537 (6)	0.7200 (4)	0.44200 (14)	0.0173 (7)	
H12A	0.8904	0.6676	0.4335	0.021*	
C13	0.6820 (6)	0.8334 (4)	0.40819 (13)	0.0151 (6)	
C14	0.4794 (6)	0.9079 (4)	0.41904 (14)	0.0189 (7)	
H14A	0.4303	0.9836	0.3950	0.023*	
C15	0.3494 (6)	0.8706 (4)	0.46533 (15)	0.0201 (7)	
H15A	0.2100	0.9213	0.4728	0.024*	

### Atomic displacement parameters (Å^2^)

**Table:**

	*U*^11^	*U*^22^	*U*^33^	*U*^12^	*U*^13^	*U*^23^
Br	0.0157 (2)	0.0143 (2)	0.0329 (3)	−0.00300 (13)	0.00039 (15)	−0.00360 (12)
Cl	0.0199 (4)	0.0216 (4)	0.0206 (4)	−0.0025 (3)	0.0026 (3)	0.0022 (3)
O	0.0112 (12)	0.0199 (13)	0.0311 (12)	0.0037 (10)	0.0003 (9)	0.0024 (10)
C1	0.0071 (14)	0.0153 (15)	0.0173 (14)	0.0035 (12)	0.0011 (10)	−0.0007 (12)
C2	0.0191 (17)	0.0126 (15)	0.0187 (15)	0.0031 (13)	0.0017 (12)	−0.0013 (12)
C3	0.027 (2)	0.0180 (18)	0.0232 (16)	0.0062 (14)	0.0053 (14)	0.0054 (13)
C4	0.0289 (19)	0.030 (2)	0.0177 (15)	0.0130 (17)	−0.0004 (13)	0.0023 (14)
C5	0.0163 (17)	0.0297 (19)	0.0227 (16)	0.0080 (15)	−0.0047 (13)	−0.0056 (14)
C6	0.0130 (16)	0.0191 (17)	0.0255 (16)	0.0005 (13)	−0.0020 (12)	−0.0024 (13)
C7	0.0125 (15)	0.0094 (14)	0.0230 (15)	−0.0002 (12)	−0.0031 (11)	−0.0009 (12)
C8	0.0149 (16)	0.0143 (15)	0.0196 (15)	0.0009 (13)	−0.0029 (12)	0.0023 (12)
C9	0.0097 (15)	0.0122 (14)	0.0197 (14)	−0.0037 (12)	−0.0027 (11)	0.0012 (12)
C10	0.0113 (15)	0.0124 (15)	0.0190 (14)	−0.0022 (12)	−0.0030 (11)	−0.0017 (12)
C11	0.0143 (16)	0.0150 (15)	0.0207 (15)	−0.0005 (13)	−0.0045 (12)	0.0033 (12)
C12	0.0118 (15)	0.0161 (16)	0.0240 (15)	0.0016 (13)	−0.0021 (12)	−0.0005 (13)
C13	0.0121 (15)	0.0156 (16)	0.0176 (14)	−0.0046 (13)	−0.0019 (11)	−0.0022 (12)
C14	0.0186 (18)	0.0148 (15)	0.0232 (15)	0.0017 (14)	−0.0038 (12)	0.0030 (13)
C15	0.0187 (17)	0.0166 (17)	0.0249 (16)	0.0045 (14)	−0.0032 (13)	0.0011 (13)

### Geometric parameters (Å, °)

**Table:**

Br—C2	1.910 (3)	C8—C9	1.334 (5)
Cl—C13	1.739 (3)	C8—H8A	0.9500
O—C7	1.219 (4)	C9—C10	1.466 (4)
C1—C2	1.388 (5)	C9—H9A	0.9500
C1—C6	1.392 (4)	C10—C11	1.389 (5)
C1—C7	1.506 (4)	C10—C15	1.402 (5)
C2—C3	1.389 (5)	C11—C12	1.395 (5)
C3—C4	1.378 (6)	C11—H11A	0.9500
C3—H3A	0.9500	C12—C13	1.389 (5)
C4—C5	1.387 (6)	C12—H12A	0.9500
C4—H4A	0.9500	C13—C14	1.384 (5)
C5—C6	1.395 (5)	C14—C15	1.383 (5)
C5—H5A	0.9500	C14—H14A	0.9500
C6—H6A	0.9500	C15—H15A	0.9500
C7—C8	1.464 (4)		
			
C2—C1—C6	118.5 (3)	C7—C8—H8A	117.1
C2—C1—C7	122.6 (3)	C8—C9—C10	126.2 (3)
C6—C1—C7	118.6 (3)	C8—C9—H9A	116.9
C1—C2—C3	121.2 (3)	C10—C9—H9A	116.9
C1—C2—Br	120.6 (2)	C11—C10—C15	118.2 (3)
C3—C2—Br	118.3 (3)	C11—C10—C9	120.0 (3)
C4—C3—C2	119.4 (3)	C15—C10—C9	121.7 (3)
C4—C3—H3A	120.3	C10—C11—C12	121.5 (3)
C2—C3—H3A	120.3	C10—C11—H11A	119.3
C3—C4—C5	121.0 (3)	C12—C11—H11A	119.3
C3—C4—H4A	119.5	C13—C12—C11	118.5 (3)
C5—C4—H4A	119.5	C13—C12—H12A	120.7
C4—C5—C6	118.9 (3)	C11—C12—H12A	120.7
C4—C5—H5A	120.5	C14—C13—C12	121.3 (3)
C6—C5—H5A	120.5	C14—C13—Cl	119.3 (3)
C1—C6—C5	121.0 (3)	C12—C13—Cl	119.4 (3)
C1—C6—H6A	119.5	C15—C14—C13	119.2 (3)
C5—C6—H6A	119.5	C15—C14—H14A	120.4
O—C7—C8	120.9 (3)	C13—C14—H14A	120.4
O—C7—C1	119.7 (3)	C14—C15—C10	121.2 (3)
C8—C7—C1	119.4 (3)	C14—C15—H15A	119.4
C9—C8—C7	125.7 (3)	C10—C15—H15A	119.4
C9—C8—H8A	117.1		
			
C6—C1—C2—C3	1.2 (5)	O—C7—C8—C9	−169.4 (3)
C7—C1—C2—C3	−172.9 (3)	C1—C7—C8—C9	11.8 (5)
C6—C1—C2—Br	−177.0 (2)	C7—C8—C9—C10	179.4 (3)
C7—C1—C2—Br	8.8 (4)	C8—C9—C10—C11	−177.2 (3)
C1—C2—C3—C4	0.8 (5)	C8—C9—C10—C15	4.7 (5)
Br—C2—C3—C4	179.0 (3)	C15—C10—C11—C12	1.4 (5)
C2—C3—C4—C5	−2.1 (6)	C9—C10—C11—C12	−176.8 (3)
C3—C4—C5—C6	1.3 (5)	C10—C11—C12—C13	0.4 (5)
C2—C1—C6—C5	−2.0 (5)	C11—C12—C13—C14	−2.0 (5)
C7—C1—C6—C5	172.4 (3)	C11—C12—C13—Cl	177.4 (2)
C4—C5—C6—C1	0.7 (5)	C12—C13—C14—C15	1.7 (5)
C2—C1—C7—O	60.4 (4)	Cl—C13—C14—C15	−177.7 (3)
C6—C1—C7—O	−113.7 (4)	C13—C14—C15—C10	0.2 (5)
C2—C1—C7—C8	−120.8 (3)	C11—C10—C15—C14	−1.7 (5)
C6—C1—C7—C8	65.1 (4)	C9—C10—C15—C14	176.5 (3)

### Hydrogen-bond geometry (Å, °)

**Table:**

*D*—H···*A*	*D*—H	H···*A*	*D*···*A*	*D*—H···*A*
C14—H14A···O^i^	0.95	2.44	3.319 (4)	154

Symmetry codes: (i) −*x*, −*y*+2, −*z*+1.

## References

[bb1] Allen, F. H., Kennard, O., Watson, D. G., Brammer, L., Orpen, A. G. & Taylor, R. (1987). *J. Chem. Soc. Perkin Trans. 2*, pp. S1–19.

[bb2] Dhar, D. N. (1981). *The Chemistry of Chalcones and Related Compounds* New York: John Wiley.

[bb3] Dimmock, J. R., Elias, D. W., Beazely, M. A. & Kandepu, N. M. (1999). *Curr. Med. Chem.* **6**, 1125–1149. 10519918

[bb4] Fun, H.-K., Patil, P. S., Dharmaprakash, S. M. & Chantrapromma, S. (2008). *Acta Cryst.* E**64**, o1464. 10.1107/S1600536808020795 PMC2962094 21203178

[bb5] Jasinski, J. P., Butcher, R. J., Narayana, B., Veena, K. & Yathirajan, H. S. (2009). *Acta Cryst.* E**65**, o2641–o2642. 10.1107/S1600536809037805 PMC2971255 21578256

[bb6] Jasinski, J. P., Butcher, R. J., Narayana, B., Veena, K. & Yathirajan, H. S. (2010). *Acta Cryst.* E**66**, o158. 10.1107/S1600536809053446 PMC2980037 21580047

[bb7] Li, H., Kamath, K. P., Narayana, B., Yathirajan, H. S. & Harrison, W. T. A. (2009). *Acta Cryst.* E**65**, o1915. 10.1107/S1600536809027615 PMC2977300 21583601

[bb8] Ng, S.-L., Razak, I. A., Fun, H.-K., Shettigar, V., Patil, P. S. & Dharmaprakash, S. M. (2006). *Acta Cryst.* E**62**, o2175–o2177.

[bb9] Oxford Diffraction (2007). *CrysAlis PRO* and *CrysAlis RED* Oxford Diffraction Ltd, Abingdon, England.

[bb10] Sarojini, B. K., Narayana, B., Ashalatha, B. V., Indira, J. & Lobo, K. J. (2006). *J. Cryst. Growth*, **295**, 54–59.

[bb11] Sheldrick, G. M. (2008). *Acta Cryst.* A**64**, 112–122. 10.1107/S0108767307043930 18156677

[bb12] Teh, J. B.-J., Patil, P. S., Fun, H.-K., Satheesh, Y. E., Razak, I. A. & Dharmaprakash, S. M. (2007). *Acta Cryst.* E**63**, o1844–o1845.

[bb13] Troeberg, L., Chen, X., Flaherty, T. M., Morty, R. E., Cheng, M., Springer, H. C., McKerrow, J. H., Kenyon, G. L., Lonsdale-Eccles, J. D., Coetzer, T. H. T. & Cohen, F. E. (2000). *Mol. Med.* **6**, 660–669. PMC1949976 11055585

[bb14] Yang, W., Wang, L. & Zhang, D. (2006). *J. Chem. Crystallogr.* **36**, 195–198.

